# Influence of multiple global change drivers on plant invasion: Additive effects are uncommon

**DOI:** 10.3389/fpls.2022.1020621

**Published:** 2022-11-14

**Authors:** Bin Yang, MiaoMiao Cui, YiZhou Du, GuangQian Ren, Jian Li, CongYan Wang, GuanLin Li, ZhiCong Dai, Susan Rutherford, Justin S. H. Wan, DaoLin Du

**Affiliations:** ^1^ Institute of Environment and Ecology, Academy of Environmental Health and Ecological Security, School of the Environment and Safety Engineering, Jiangsu University, Zhenjiang, China; ^2^ School of Computer Science, Faculty of Engineering, University of Sydney, Darlington, NSW, Australia

**Keywords:** climate change, nitrogen deposition, precipitation, temperature, effect sizes, interactions

## Abstract

Invasive plants threaten biodiversity and cause huge economic losses. It is thought that global change factors (GCFs) associated with climate change (including shifts in temperature, precipitation, nitrogen, and atmospheric CO_2_) will amplify their impacts. However, only few studies assessed mixed factors on plant invasion. We collated the literature on plant responses to GCFs to explore independent, combined, and interactive effects on performance and competitiveness of native and invasive plants. From 176 plant species, our results showed that: (1) when native and invasive plants are affected by both independent and multiple GCFs, there is an overall positive effect on plant performance, but a negative effect on plant competitiveness; (2) under increased precipitation or in combination with temperature, most invasive plants gain advantages over natives; and (3) interactions between GCFs on plant performance and competitiveness were mostly synergistic or antagonistic. Our results indicate that native and invasive plants may be affected by independent or combined GCFs, and invasive plants likely gain advantages over native plants. The interactive effects of factors on plants were non-additive, but the advantages of invasive plants may not increase indefinitely. Our findings show that inferring the impacts of climate change on plant invasion from factors individually could be misleading. More mixed factor studies are needed to predict plant invasions under global change.

## Highlights

1. The effects of global change factors on plant invasion are interactive, and additive effects are uncommon.
2. The performance and competitiveness of invasive plants may benefit from the impact of global change.
3. Plant performance and competitiveness changes are not always in the same direction.

## Introduction

Numerous plant species are being introduced into new habitats from their original habitats intentionally or unintentionally by humans. Many of these have adapted to their new habitats and become naturalized, and some have become invasive, resulting in the negative effects on the structure and function of local ecosystems (Kumar Rai and Singh, 2020; Pyšek et al., 2020). Ecologists are increasingly paying more attention to the field of invasion biology due to the increasing harm of plant invasions (van Kleunen et al., 2018). It is generally believed that invasive alien plants are successful because that they have broader environmental tolerance (Scasta et al., 2015; Liao et al., 2019), higher phenotypic plasticity (Richards et al., 2006; Scasta et al., 2015), and resource utilization capacity (Wang et al., 2013) than native plants. These traits may help them adapt to new environments through performance and competitive advantages (Kuebbing and Nuñez, 2016).

As a complex ecological process, plant invasion is affected by many biotic (e.g. phenotypic traits, soil microorganisms, enemy release; Keane and Crawley, 2002; Gosper, 2004; Grotkopp and Rejmanek, 2007; Zhang et al., 2020) and abiotic factors (e.g. temperature, humidity, soil nutrient content; Yu et al., 2018; Liu et al., 2018a; Ren et al., 2020). Global change factors (GCFs) including temperature increase, rainfall variability, nitrogen deposition, and atmospheric carbon dioxide (CO_2_) enrichment can moderate the performance and competitive interactions between native and invasive plants, which can affect invasion success (Liu et al., 2017; Wu et al., 2017; Shrestha and Shrestha, 2019). It is generally believed that changes in these factors have created new environments (e.g. by changing the availability of resources; Jia et al., 2016), while the intrinsic traits of invasive plants may allow them to take advantage of their new environment (van Kleunen et al., 2010), thus they can obtain better performance than native plants under global change (Dostál et al., 2013). A lot of experimental studies have been conducted all over the world to examine how plant invasions will change in response to GCFs, especially to many factors independently (Jia et al., 2016; Liu et al., 2017; Stephens et al., 2019).

**Figure 1:**
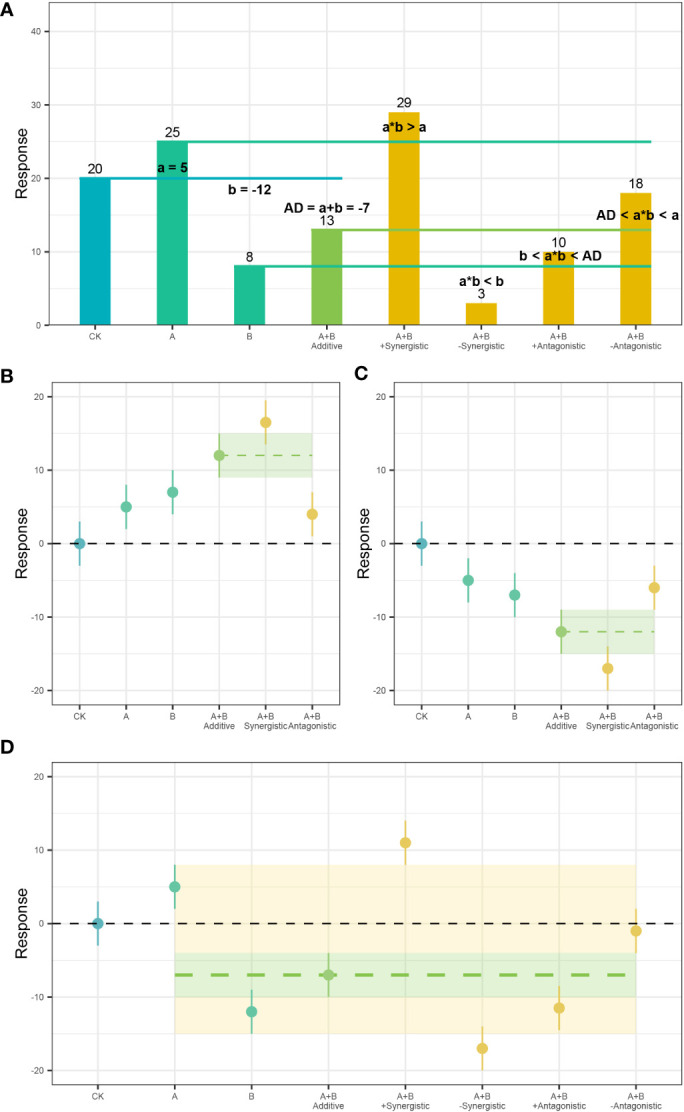
An illustration of the system for classifying plant responses to multiple global change factors based on the direction and magnitude of their individual and combined effects. According to the interaction type of two factors with double positive, double negative and opposite individual effects (see Figure 1 of Crain et al. 2008), the interaction type **(A)** of “limiting synergy” proposed by Piggott et al. (2015a) under opposite individual effects. In this study, interaction classification criteria for double positive **(B)**, double negative **(C)** and opposite **(D)** individual effects. In the figure, bold numbers denote net trait response from factor A, B or A+B, numbers in brackets denote antagonistic or synergistic effects.

Of course, GCFs occur simultaneously in nature and different GCFs have interactive effects on ecosystem processes (Reich et al., 2006; Li et al., 2016; Yue et al., 2017; Yue et al., 2019; Orr et al., 2022). Although the terms “synergistic” and “antagonistic” are often used to describe the possible interactions between multiple drivers, there is still a lack of consensus on definitions (Piggott et al., 2015a; Hale et al., 2017; Orr et al., 2022). In this study, an additive effect (i.e., no interaction between factors) is defined as the case where the combined effects of two or more factors are equal to the sum of those effects individually (Colby, 1967; Rummens, 1975; Crain et al., 2008). In contrast synergistic or antagonistic interactions between factors include cases where the combined effects can produce results that are significantly different or exaggerated than is expected (Gurevitch et al., 2000; Yue et al., 2019). The definition used by Crain et al. (2008) has been widely cited and relatively well recognized. According to the direction of the independent effect of the two factors, it can be divided into two scenarios: If the individual effects of each factor are in the same direction (either positive or negative), a synergistic effect is defined as the case where the combination of two factors is stronger than the sum of effects independently. For example, factor A and factor B both inhibit the growth of plants. In combination, A will prompt B to strengthen its inhibitory effect on plants (Figure 1 of Crain et al. 2008). Another situation is that the factors have opposing effects (e.g. independently, A is positive but B is negative). In this case, the classification of the interaction between the two factors is controversial. Gurevitch et al. (2000); Crain et al. (2008); Rillig et al. (2019) and others stated that if the combined effect of the two factors is more negative than the cumulative effect of the two factors, it is considered a synergistic effect. On the contrary, it is an antagonistic effect where the combined effect produces a more negative response than what it should be given an additive effect (Figure 1 of Crain et al. 2008). In a series of articles, Piggott et al. (2015a; 2015b; 2015c) proposed a classification system based on the additive effect model, which further refined the classification of interactions, and a new class of “mitigating synergism” is identified to accumulate the magnitude of the effect is measured compared with the individual stress effect to clarify the interaction effect of two factors with opposite effects (**Figure 1A**), and has been used by some authors recently (Hale et al., 2017; Cremona et al., 2021; Jin et al., 2021).

If the interaction between GCFs is additive, the effects of multiple GCFs on plant invasion can be inferred from the effects of single GCFs individually. But in reality, the effects from the interactions between GCFs are highly variable. For example, there is an interaction effect of nitrogen deposition and water availability on the response of the invasive herb *Bromus madritensis*, and its sensitivity to soil nitrogen depends on the degree of water limitation, but there is no interaction between the two GCFs (Rao and Allen, 2010), indicating that the promoting effect of nitrogen deposition on plant invasion would intensify with an increase in precipitation. In addition, the benefit to invasive plants from nitrogen deposition may increase as temperatures rise. Ren et al. (2020) studied phenotypic plasticity and fitness of invasive *Solidago canadensis* under variable nitrogen and temperature, and found that warming and increased nitrogen would create conditions more suitable for growth, and may increase invasion risk by enhancing phenotypic plasticity. In another study, increases in CO_2_ concentration and temperature promoted the aboveground growth of the native *Austrodanthonia eriantha* and the invasive *Vulpia myuros*, respectively (Hely and Roxburgh, 2005). However, the combined treatment of temperature and CO_2_ had a limited effect on growth promotion, and the degree of enhancement was lower than that expected (versus the sum of the effects from each factor individually). To an extent, temperature and CO_2_ concentration mutually inhibited each other’s promoting effect on plants (Hely and Roxburgh, 2005). In Larson et al. (2018), drought stress enhanced the inhibitive effect of native *Pseudoroegneria spicata* on the invasive *Bromus tectorum* under high CO_2_ conditions. A better understanding for more accurate predictions on how plant invasions behave under global change is crucial (Gioria and Osborne, 2014). Currently the interaction between the driving effects of multiple GCFs on plant invasion is often extrapolated from single factor effects. There is some feasibility in predicting the combined effects from multiple factors, but there is a high level of uncertainty. Therefore, a synthesis of existing research results to explore the combined effects of multiple GCFs on plant invasion is necessary.

Currently, research on plant invasion often focuses on the performance of plants and on the biotic interactions between plants (e.g. competition). But the current comprehensive studies (Jia et al., 2016; Liu et al., 2017; Stephens et al., 2019) mainly focus on the performance of plants growing individually (and not under competitive conditions). A focus on competition is important because a stronger competitiveness may help invasive plants occupy niches originally occupied by local indigenous communities (Barton and Wong, 2019). Competitive ability against native plants may be a key factor that determines whether invasive plants can successfully invade (Gioria and Osborne, 2014; Zhang and van Kleunen, 2019). GCFs often have significant effects on the competition between invasive and native plants, but the effects of multiple GCFs are not well understood. Although increased CO_2_ may not drastically change the competitive interaction between native and invasive plants, it may increase the comparative advantage of invasive plants over native plants (Manea and Leishman, 2011). Nitrogen deposition also promoted the competitive ability of invasive plants through increasing root biomass (Broadbent et al., 2018). The relative performance non-naturalized alien garden plants may increase under combined warming and precipitation change, as resident species become less competitive (Haeuser et al., 2019). The differences in interaction between different combinations of factors indicate that a comprehensive study on the interactive effect of multiple GCFs on plant invasion is necessary for better predictions. A quantitative synthesis of the data across many studies is needed to predict the overall development trend of plant invasion under the background of global change (Jia et al., 2016).

Here, we synthesized the data from the published literature on the effects of GCFs on plant invasion. The main objectives of this study are to better understand the effects of major GCFs on independent performance and competitiveness of native and invasive plants, and to investigate whether the effects of independent GCFs on plant invasion are additive. We hypothesize that: (1) Native and invasive plants will show different responses in performance and competitiveness to each other under GCFs, and invasive plants can benefit more from the effect; and (2) there is a significant difference between the combined effects (of any two GCFs on native and invasive plants) and the sum of their independent effects, and it will be an antagonistic effect (sub-additive effect).

## Materials and methods

### Data compilation

To identify studies reporting the responses of native and invasive plants to global changes, we searched the ISI Web of Science (http://apps.webofknowledge.com/) using the words related to “global change”, “plant invasion”, and “competition” as keywords. A total of 6,509 papers published before May 2020 was assessed. Each publication was individually assessed and retained if: 1) The research data were obtained through plant competition experiments, and at least one treatment group was the competition between native and invasive plants at the study location; and 2) in the competitive experiment, at least one global change factor treatment was applied. We regard the treatment of deviation from normal environmental values in the following environmental variables as GCFs. GCFs include temperature increase (T), precipitation increase (P), drought intensification (D), nitrogen deposition (N), and atmospheric CO_2_ enrichment (C). It is worth noting that although both P and D involve changes in water availability, they involve different types of stressors on plants with the former involving waterlogging and disturbance and the latter involving low soil water potential (Liu et al., 2017; Song et al., 2019; Zhou et al., 2020). Thus, P and D were analyzed separately.

We directly extracted the data from the text and table of the main body and appendices of these papers. The GetData Graph Digitizer (version 2.24, available online: http://getdata-graph-digitizer.com/) was used to extract the data from the figures. The following criteria were applied to extract data from each study: (1) The studies were divided into two categories according to the competitive treatment employed; that is, the relative neighbor effect (RNE) and relative competition effect (RCE) depending on the measurement reported by the study (Kuebbing and Nuñez, 2016). RNE measures the competitive ability of the target species by comparing the relative difference between the species grown alone and under competition with a neighboring plant(s). RCE measures the competitiveness of the target plant by comparing its relative performance with that of competing plants in mixed planting. Usually, RNE data is inclusive of RCE, however, RCE may not be attainable in studies reporting RNE. Moreover, co-occurring native and invasive plants may compete for resources. Therefore, the results of our study only kept the plant performance data under mixture of native and invasive plants. The invasive plants were considered the target plants to study the competitiveness of invasive plants against native plants (the competing plants), and vice versa for the competitiveness of native plants. (2) To minimize differences in methods for calculating competitiveness among studies, we gave priority to studies reporting plant performance (i.e., growth performance, physiological performance, survivorship performance) and directly recorded the competitiveness results for studies that only disclose plant competitiveness. Growth performance traits include biomass or size, physiological performance traits mainly refer to photosynthesis, which can directly increase the performance of plants, and survivorship traits include survival or reproduction. (3) We considered the ambient level of an environmental change factor (i.e., precipitation, temperature, atmospheric CO_2_ concentration, and soil N) as the control and elevated level of the same factor as the treatment. If the paper did not specify which level is the ambient level, the treatment with the lowest value was considered the control. An exception to this was for the drought treatments, where the normal water level was taken as the ambient level, and the drought treatment was taken as the treatment. Compared with previous meta-analyses (Sorte et al., 2013; Jia et al., 2016; Liu et al., 2017; Stephens et al., 2019), we chose to retain the multiple factor results and used it as an important classification criterion in the analysis. Different multiple factor studies are collected as different groups of GCFs. (4) If the research across a period of time, only the final data point was extracted to avoid oversampling of data.

### Statistical analysis

To study the effects of global change factors on the performance and competitiveness of native and invasive plants, we analyzed the plant performance data and the competitiveness of each plant. We standardized the plant performance data under competition using the Relative Competition Intensity index (RCI; Weigelt and Jolliffe, 2003) defined by the following formula:


(Equation 1) $$RCI=\frac{P_{t}-P_{c}}{P_{c}}$$

where *P_t_* and *P_c_* are the mean standardized data of the target plant and competing plant performance, respectively; and *P* is defined by:


(Equation 2) $$P=\frac{p_{o}}{\left|p_{ck}\right|+\left|p_{e}\right|}$$

where *P* is the corresponding standardized data, *p_o_* is original performance of target or competing plant under control or experimental treatment, and *p_ck_* is original plant performance under control treatment, and *p_e_* is under experimental treatment.

**Figure 2:**
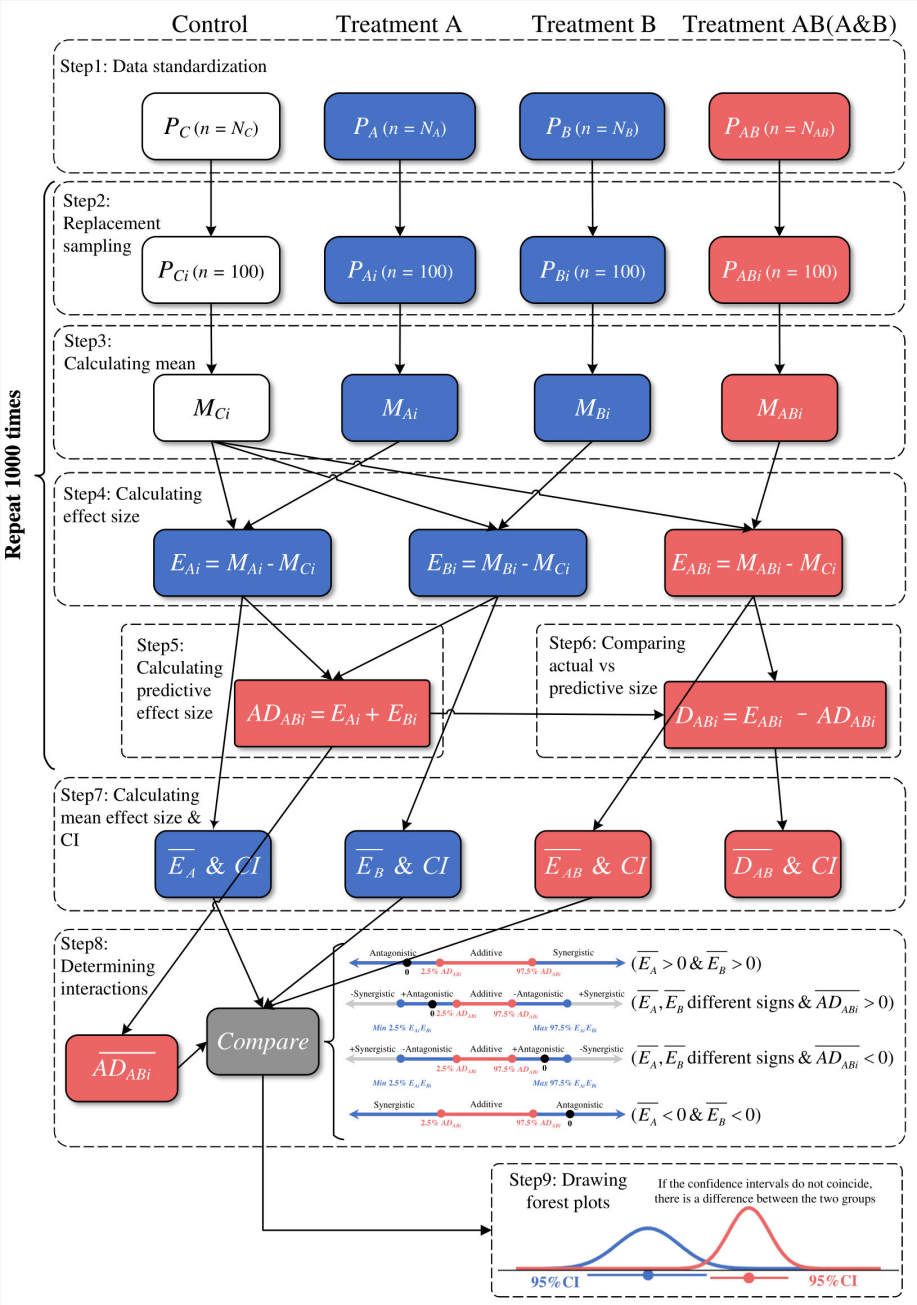
Schematic of the bootstrap resampling design for estimating the joint effect sizes of single and multiple GCFs treatments on performance and competitiveness of native and invasive plants using plant performance as an example. In step 1, according to no treatment (*P_C_*, sample size of data is *N_C_*), A factor alone (*P_A_*, sample size of data is *N_A_*), B factor alone (*P_B_*, sample size of data is *N_B_*) and AB two-factor interaction (*P_AB_*, sample size of data is *N_AB_*) standardized plant performance data. From steps 2 to 6, it is repeated 1000 times, and the number of iterations is marked with *i* in subscript form. In step 2, 100 samples from *P_C_*, *P_A_*, *P_B_* and *P_AB_* were randomly placed back to draw each as *P_Ci_*, *P_Ai_*, *P_Bi_* and *P_ABi_*, and in step 3, the mean values of 100 random samples were calculated as *M_Ci_*, *M_Ai_*, *M_Bi_* and *M_ABi_*, the single A factor (E*_Ai_*), the single B factor (*E_Bi_*) and the A, B two-factor combination (*E_ABi_*) are calculated in step 4 by calculating the difference between the average value under the obtained treatment and the average value of the control group. Effect size for *i* iterations. Step 5, estimate the predicted two-factor additive effect size (*AD_ABi_*) based on the effect size of the single factors A and B, and compare the difference between the actual effect size of the two factors A and B and the predicted additive value (*D_ABi_*) in step 6. Step 7, calculate the mean and 2.5% and 97.5% quantiles of the effect size and difference size obtained by 1000 iterations, as the lower and upper interval of the combined effect (difference) size and confidence interval. In step 8, interactions are identified according to the interaction classification criteria and plotted against the data in a final step 9.

For both performance (*P*) and competition (*RCI*) data, we used the non-parametric bootstrap method to estimate the mean effect sizes and confidence intervals (CI) for the plant performance and competitiveness data, so that we could use the intermediate results to calculate the single factor additive effect sizes. The algorithm conducts sub-sampling with replacement (1,000 iterations). This method does not emphasize statistical significance thresholds, and emphasizes comparing the mean effect sizes and 95% CIs to assess the impact of each treatment (Rillig et al., 2019). Using the process of analyzing plant performance data as an example (**Figure 2**), we first grouped the data according to GCFs, and then by random replacement 100 data points from each group was sampled and the mean value was calculated. We defined the effect sizes of single and multiple GCFs as the raw mean differences in sample means between the “control” group and the “treatment” group, and directly used the original difference between them to evaluate the difference size between the actual multiple factor effects and the additive effects of corresponding single GCF (Rillig et al., 2019). The above steps were repeated a total of 1,000 times. Each group comprised 1,000 effect sizes (single and multiple GCFs) or 1,000 difference sizes (only multiple GCFs) used to calculate the corresponding mean effect sizes. The quantiles of 2.5% and 97.5% were used as the lower and upper limits of 95% confidence intervals. *RCI* used the same method for statistical analysis. For effect sizes, the positive or negative effect size means whether the effect of GCFs is positive or negative. By calculating the proportion of 1000 effect sizes or difference sizes greater than 0 as p, when 0.025< *p<* 0.975 means 0 is within the CI range, indicating that the treatment effect is not significant. When 0 is within the CI, it means that the treatment has no significant effect. A significant difference is considered to be where CIs do not overlap among the two groups. Finally, we classified each result obtained from 1000 iterations separately (**Figures 1B–D**) according to the interactive classification system (A~D) of Crain et al. (2008) and Piggott et al. (2015a). The workflow for the processing of raw data is summarized in **Figure 2**. The classification is described below:

1. An *additive* response (AD) indicates that there is no interaction effect between the two single factors (relative to the control), and the combined effect of the two factors is as expected (i.e., AD or expected [a + b], as shown in **Figure 1D**). That is, it is the same as the sum of the two factor effects on the plant response individually. In **Figure 1D**, the effect of factor A on the plant response is +5 but for factor B it is -12. An additive effect is demonstrated when the expected net effect of -7 (relative to the control) is present when under the effects of both factors.
2. *Antagonistic* (A) interactions are non-additive, and appears to be weaker than expected (i.e.< a + b and< |a| or |b|). If the two factors act in opposite directions, they can be further divided into *+Antagonistic* (stronger than expected effect, +A) and *−Antagonistic* (weaker than expected effect, −A). In **Figure 1D**, a positive antagonistic effect is present when the net effect of -10 is less than the additive response (expected [a + b] = -7), but still greater than the factor with the more negative effect on plant response (in this case, factor b = -12). A negative antagonistic effect is present when the net effect of -2 (absolute response of 18) is greater than the expected additive effect of -7, but lesser than the factor with the greater response (in this case, a = 5).
3. *Synergistic* (S) interactions are non-additive, and appears to be stronger than expected (> a + b and > |a| or |b|). If the two factors act in opposite directions, they can be further divided into +*Synergistic* (weaker than expected effect, +S) and −*Synergistic* (stronger than expected effect, −S). In **Figure 2D**, a positive synergistic response is present when the net effect of 9 is greater than the factor with a higher effect (a = 5 in this case). A negative synergistic response is present when the net effect (-17) is lower than the lesser response among the two factors (b = -12 in this case).

All analyses were conducted using R (version 4.2.0, R Core Development Team) using the “xlsx”, “plyr”, “data.table” and “stringr” packages, and the forest plots were generated using “ggplot2” and “ggpubr”. Flowcharts are drawn using Microsoft Visio 2019 (Microsoft Corp., Redmond, MA, USA).

## Results

We retrieved 30 case studies that included 176 plant species (including 101 native and 76 invasive species; **Figure S1**). One of the species had different identities in different studies (*Ipomoea pes-caprae*). There were 340 data points (296 single and 44 multiple GCF) on performance and/or competitiveness of native and invasive plants (**Figure S2**; **Appendix S1**; **Table S1**). We found that only a small number of studies involving two factors, and not all combinations were available. For the study of more than two factors, we only found one study with a 3-way interaction (i.e., warming × nitrogen deposition × CO_2_ enrichment; Larson et al., 2018). Because of the lack of studies testing three factors, our discussion on multiple GCFs effects here mainly involves combinations of two factors.

### Single GCFs independent effects

**Figure 3:**
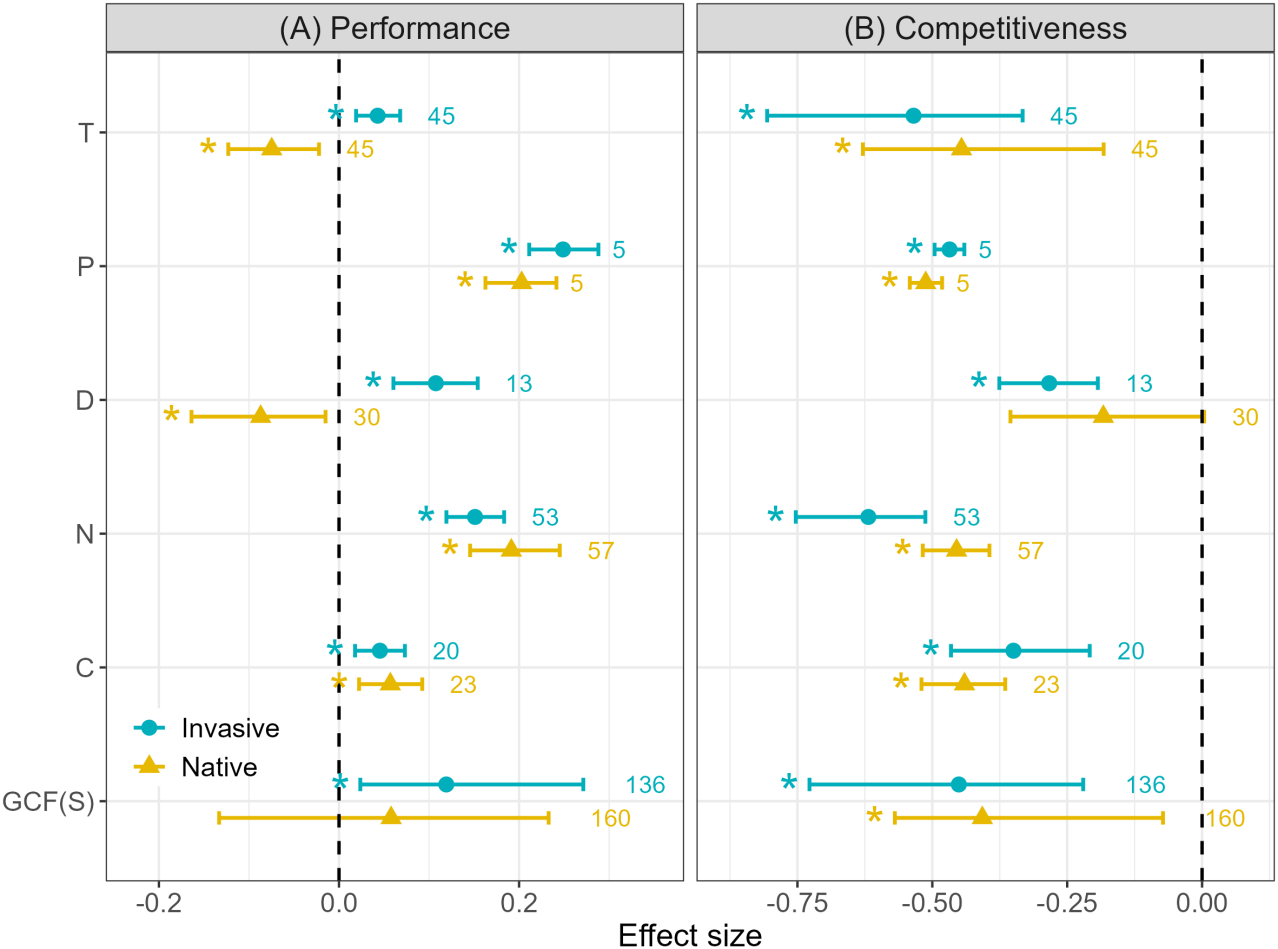
Independent effects of single GCFs on plant invasion in: **(A)** plant performance (left panel) and **(B)** competitiveness (right panel). Values indicate the means with 95% confidence intervals (CIs), and original sample size numbers for native and invasive plants are shown on the right hand side of dots. The * on the left of the means indicates the effect size is significantly different from zero (CI does not overlap with 0). A significant positive effect is where the CI is greater than 0 and a negative effect is where the CI is less than 0. Factors represented in the ordination: T = temperature increase, P = precipitation increase, D = drought intensification, N = nitrogen deposition, C = atmospheric carbon dioxide enrichment, GCFs (S) = single global change factors summary

We found that single GCFs had an overall positive effect on the performance of invasive plants (0.234, *p* = 1.000 > 0.975, **Figure 3**). For native plants, although GCFs also had a positive effect on the whole (0.058, *p* = 0.602), the positive effect of GCF on native plants was not significant due to the adverse effects of temperature increase and drought on the growth of native plants (−0.075, p = 0.005< 0.025 and −0.164, *p* = 0.008< 0.025 respectively). Among the GCFs, precipitation increase had the most positive effect on native and invasive plants (0.203, *p* = 1.000 > 0.975 and 0.249, *p* = 1.000 > 0.975 respectively). The biggest difference in response between native and invasive plants was in the effect of drought (native: −0.087, *p* = 0.008< 0.025, invasive: 0.108, *p* = 1.000>0.975), and the smallest difference was in atmospheric CO_2_ enrichment (native: 0.057, *p* = 0.999 > 0.975, invasive: 0.045, *p* = 1.000 > 0.975). On the whole, single GCFs have a negative impact on the competitiveness of native and invasive plants, and the negative impact on the competitiveness of invasive plants is slightly greater than that of native plants (native: −0.407, *p* = 0.007< 0.025, invasive: −0.451, *p* = 0.000< 0.025, **Figure 3**). Overall, the negative impact of single GCFs on the competitiveness of invasive plants was also far less than that of native plants (−0.022 ± 0.017 and −0.462 ± 0.015 respectively). The results showed that except drought had no significant effect on the competitiveness of native plants (−0.183, *p* = 0.030), other single GCFs had significant negative effects on the competitiveness of native and invasive plants (p< 0.025).

### Multiple GCFs combined effects

**Figure 4:**
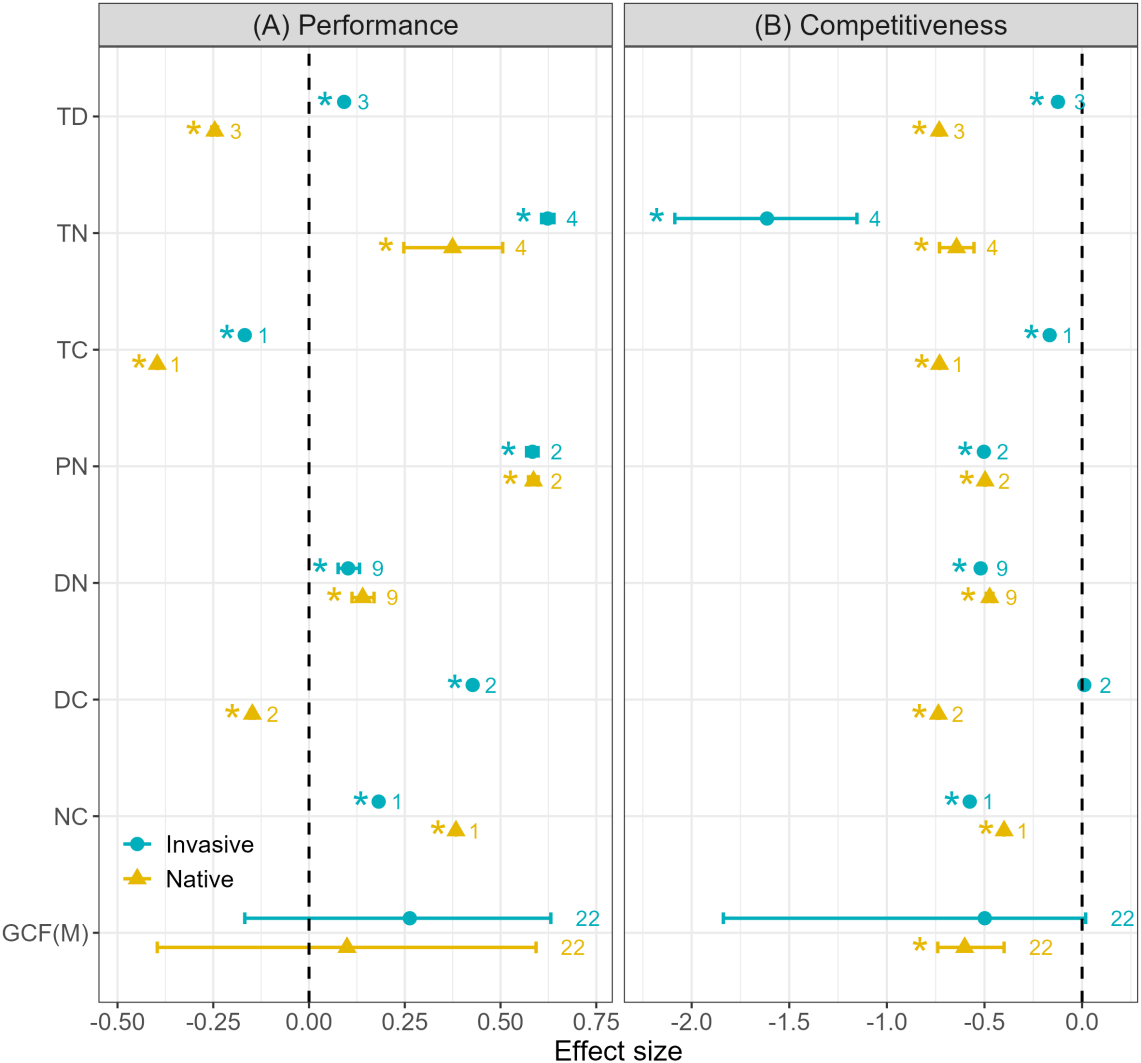
Combined effects of multiple GCFs on plant invasion in **(A)** plant performance (left panel) and **(B)** competitiveness (right panel). Values indicate the means with 95% confidence intervals (CIs), and original sample size numbers for native and invasive plants are shown right the dots. The * to the left of the dots indicates that CI does not overlap zero, which means that the GCF has a significant impact on plants, and positive effect is where the CI is greater than zero, or a negative effect where the CI is lower than zero. TD: temperature increase × drought intensification, TN: temperature increase × nitrogen deposition, TC: temperature increase × atmospheric carbon dioxide enrichment, PN: precipitation increase × nitrogen deposition, DN: drought intensification × nitrogen deposition, DC: drought intensification × atmospheric carbon dioxide enrichment, NC: nitrogen deposition × atmospheric carbon dioxide enrichment, GCFs(M): multiple global change factors summary.

Most multiple GCFs combinations had positive effects on plant performance, and the positive effect on invasive plants was slightly greater than that on native plants (native: 0.099, *p* = 0.571, invasive: 0.263, *p* = 0.857, **Figure 4**). Under the influence of most multiple GCFs, the response direction of native and invasive plants is the same, especially temperature increase × nitrogen deposition (TN, native: 0.375, *p* = 1.000 > 0.975, invasive: 0.623, *p* = 1.000< 0.975) and drought intensification × nitrogen deposition (DN, native: 0.140, *p* = 1.000 > 0.975, invasive: 0.102, *p* = 1.000< 0.975). And a small number of GCFs combinations have opposite effects on the performance of native and invasive plants. For example, temperature increase × drought intensification (TD, native: -0.246, *p* = 0.000 > 0.025, invasive: 0.092, *p* = 1.000< 0.975) can promote the performance of invasive plants, but inhibit the performance of native plants. For the effects of GCFs on plant competitiveness, multiple GCF combinations usually have negative effects (native: -0.601, *p* = 0.000 > 0.025, invasive: -0.499, *p* = 0.134, **Figure 4**). All GCFs combinations reduced the RCI of native plants, and only drought intensification × atmospheric CO_2_ enrichment (DC) slightly promoted the competitiveness of invasive plants (0.011, *p* = 0.03 > 0.025).

### Multiple GCFs interactive effects

**Figure 5:**
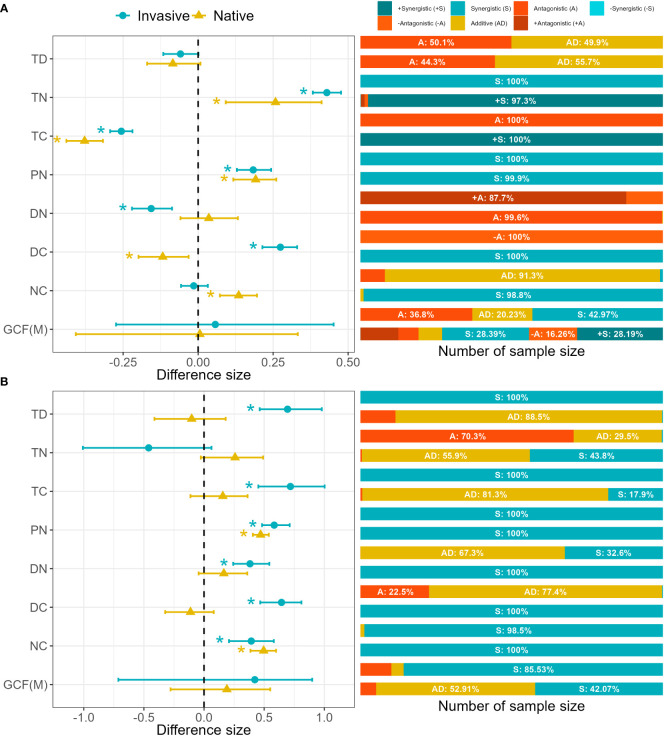
Interactive effects of multiple GCFs on invasive plants: **(A)** plant performance and **(B)** competitiveness. Values indicate the means with 95% confidence intervals (CIs) in left panel. The * to the left of the dots indicates that CI does not overlap with zero meaning that the GCF has a significant interaction effect on plants. Values in percentages in indicate the proportions of the type of interaction effect among factors. Additive effects are cases where the effect under multiple GCFs is the same as the sum of those effects individually. Antagonistic and synergistic effects (unsigned) are cases where both factors have effects in the same direction, while signed effects (positive and negative) are weaker or stronger than expected. Details on classifications of these interactive effects are in the text. The abbreviations for the factors are the same as those in **Figure 5**.

In general, the combined interaction of multiple GCFs on plant performance is additive (native: 0.006, *p* = 0.543; invasive: 0.057, *p* = 0.471, **Figure 5A**), but this is due to the significant and opposite interaction of different combinations, as for the interaction results of each iteration, non-additive interactions such as antagonism and synergy seem to be more common than additive interactions (native: 91.85% and invasive: 79.77% are non-additive),. In terms of the response results of native plants, all of them were non-additive interactions except that TD (-0.084, *p* = 0.044) had a 55.7% possibility of additive effects. TN (0.258, *p* = 0.999>0.975, S: 100%), precipitation increase × nitrogen deposition (PN, 0.192, *p* = 1.000 > 0.975, S:99.9%), DC (-0.118, *p* = 0.004< 0.025, S: 100%) and NC (0.135, *p* = 1.000 > 0.975, S: 98.8%) are (+)synergistic effects, while DN (0.036, *p* = 0.757, A: 99.6%) was antagonistic effect. In the response results of invasive plants, only the combination of TD (−0.059, *p* = 0.031, AD (Additive effect): 49.9%) and NC (−0.015, *p* = 0.265, AD: 91.3%) were great possibility of additivity. While in the non-additive interaction, TN (0.429, *p* = 1.000 > 0.975, S: 100%) and (0.184, *p* = 1.000>0.975, S: 100%), were synergistic, while TC (−0.256, *p* = 0.000< 0.025, A: 100%), DN (−0.156, *p* = 0.000<0.025, +A&-A: 100%), and DC (0.274, *p* = 1.000>0.975, -A: 100%) were (+/-) antagonistic. In other words, most of the interaction effects of seven multiple GCF combinations on native and invasive plants were non-additive (85.81%, **Figure 5A**). The interactive effects of various GCFs on plant competition between native plants and invasive plants are completely different, and the interactive responses to multiple factors are mostly synergistic interactions (Mean of native and invasive: 63.80%). Among the effects on native plants, PN (0.471, *p* = 1.000 > 0.975, S: 100%), DN (0.165, *p* = 0.941, S: 100%), DC (-0.113, *p* = 0.128, S: 100%), and NC (0.496, *p* = 1.000 > 0.975, S: 100%) were synergistic, while the other three combinations were additive. However, most of the interactive effects on invasive plants were synergistic or antagonistic, and only DC (0.644, *p* = 1 > 0.975, AD: 77.4%) was additive (**Figure 5B**).

## Discussion

Our synthesis indicated that major global change factors (including warming, precipitation increase, drought intensification, nitrogen deposition and increasing atmospheric CO_2_) may affect the performance and competitiveness of invasive and native plants at different magnitudes and/or directions. Although invasive plants are more likely to benefit from shifts in GCFs in most cases, our results indicate the interactions among multiple GCFs were often non-additive and variable. This means that in the context of global change in the future, although we can basically confirm that plant invasion will further intensify, the advantages of invasive plants will not increase indefinitely with the increase in GCFs.

### Single GCFs on performance and competitiveness

We found that different single GCFs have different individual effects on the performance of native and invasive plants. For plant performance, invasive plants usually benefited more from GCFs compared to native plants. For competitiveness, invasive plants are as greatly affected as native plants, where they usually show negative responses under GCFs. On the whole, these findings partly confirm our first hypothesis that the performances of invasive plants are more favored by single GCFs than native plants.

Temperature increase and drought intensification are generally stressful for plants (Zandalinas et al., 2018), and the difference in traits between native and invasive plants determines their sensitivities to stress (van Kleunen et al., 2010). From the results, temperature increase and drought intensification tended to have opposite effects on the performance of both invasive and native plants. However, under increased resources such as precipitation increase, nitrogen deposition, and elevated CO_2_, both native and invasive plants tend to benefit. Although competition may reduce those gains, the overall responses were still positive. We found that the competitiveness of plants under the effects of independent GCFs tended to be more negative than plant performance. This suggests that the competition between native and invasive plants may gradually weaken in the global changing ecological environment (Yu et al., 2018; Azeem et al., 2022). We also found that GCFs improved the performance of plants overall, especially for invasive plants, but had a negative impact on the competitiveness of both invasive and native plants. Based on our performance and competition results, change in water regime (precipitation or drought) is most favorable for invasive plants among independent GCFs. The reason for this is unclear, but limited water availability and disturbance (including heavy rainfall events) could inhibit native plants, favoring invasive plants (Orbán et al., 2021). Increased CO_2_ could also increase the advantages of invasive plants over native plants. High CO_2_ may increase the primary productivity of native and invasive plants at the same time, but the aboveground biomass and rhizome size of invasive plants may increase more than native plants (Nackley et al., 2017; Oliveira et al., 2021). However, our results also suggest that the competitiveness of invasive species tended to be more impacted than native species. The overall result of this study is in contrast with previous views (Liu et al., 2018b; Wang et al., 2019) that invasive plants will have higher competitiveness due to global changes. Our results are more consistent with the belief that fluctuating resource availability will weaken competition (Davis et al., 2000).

We found that the response direction of native and invasive plants to GCFs may be different. It was previously thought that they would have similar responses to the same environmental change (Liu et al., 2017). Compared with the previous studies, our study also analyzed the competitive response of plants (not only in the absence of competition). Our research data were all derived from competitive experiments. In addition, the research methods are also different to previous similar research (Sorte et al., 2013; Liu et al., 2017). On one hand, to eliminate the differences between plant species, we use standardized data and take the raw difference between treatment and control as the effect size value (Wang et al., 2017; Rillig et al., 2019). On the other hand, in the process of data integration, we referred to the bootstrap method of Rillig et al. (2019) instead of the traditional meta-analysis method (Gurevitch and Hedges, 1999). Although these may lead to some differences between our results and those of previous studies, our results show that invasive plants will perform better than native plants under the influence of GCFs, which is consistent with the majority of the literature (e.g., Song et al., 2010; Pearson et al., 2017; Ren et al., 2021).

### Combined effects and the interactions of multiple GCFs

We found that invasive plants will gain advantages over native plants in terms of performance and competitiveness under the combined effects of multiple GCFs, which supports the rest of our first hypothesis (that invasive plants will benefit from global change). The GCF combination most favorable for invasive plants is CO_2_ enrichment and drought. On one hand, this combination significantly improves the performance of invasive plants and hinders the growth of native plants. On the other hand, it significantly inhibits the competitiveness of native plants. Drought can first reduce growth and kill some individuals of native plants. Then, under CO_2_ fertilization (e.g., Osborne, 2016), invasive plants can quickly occupy the resource space originally occupied by native plants, obtaining stronger relative performance and competitiveness (Manea et al., 2016). The present study also demonstrates that the interaction of multiple GCFs are usually synergistic (super-additive effect) or antagonistic (i.e., sub-additive effect), rather than additive. This confirms the first part of our second hypothesis that the effects from multiple GCFs are non-additive, but the hypothesis that it will be an antagonistic was not supported. Understanding the interaction of multiple GCFs on the performances and competitiveness of native and invasive plants is crucial for predicting the response of plant invasion to global change in the future (Shrestha and Shrestha, 2019; Rillig et al., 2019).

The interaction between multiple GCFs for invasive and native plant performance were similar. For plant performance, temperature increase and nitrogen deposition had a synergistic effect. The literature showed that increased temperature may increase biomass allocation to roots while the belowground biomass reduced slightly (Crosby et al., 2017). However, abundant nitrogen can help plants obtain sufficient nutrients with less underground parts to make up for the reduction in belowground biomass. Interestingly, the response of aboveground biomass of native and invasive plants to nutrient addition was different under elevated temperature. For example, a study found that under increased nutrients the aboveground biomass of invasive *Spartina alterniflora* increased, but the aboveground production of native *Phragmites australis* only increased slightly (Legault et al., 2018). Therefore, although both native and invasive plants may gain growth benefits from combined temperature increase and nitrogen deposition, the benefits to invasive plants may be greater. Warming can furthermore have positive effects on nitrogen fixation and/or microbial decomposers, which can further improve the availability of soil resources to plants (Convey et al., 2012).

Some multiple GCFs combinations were different among invasive versus native plants. For example, combined drought intensification and nitrogen deposition tended to be synergistic on the performance and competitiveness of native plants, but for invasive plants it tended to be antagonistic. This indicates that the intensification of drought may be more beneficial to native plants under nitrogen deposition, but the mechanism that causes this phenomenon is disputed. Some studies suggest that invasive plants have better resource utilization ability, but this is only in environments high in water availability and resources. So, drought should inhibit the beneficial effects of high nitrogen on the performance and competitiveness of invasive plants (Wei et al., 2017). Conversely, Valliere (2019) found that invasive plants can gain an advantage under the same conditions, arguing that invasive plants have more efficient trade-offs between water and resources. This inconsistency may be due to specific differences among plant species.

Only one study was included for combined precipitation increase and nitrogen deposition (Rao and Allen, 2010), who found that the degree of plant response to nitrogen addition depends on the degree of water limitation, and that both native and invasive plants are mainly restricted by water. Similarly, the sample sizes for increased temperature × elevated CO_2_, drought × elevated CO_2_, and nitrogen deposition × elevated CO_2_ were only one or two. A meta-analysis on the effect of GCFs on soil respiration also only found few (1-2) articles assessing multiple GCFs (Zhou et al., 2016). The relatively low number of studies available revealed that the effect of multiple GCFs on plant invasions is understudied.

Our results indicate that in most cases, plant responses under multiple GCFs should not be directly inferred from results based on factors tested individually (especially the impact on the competitiveness of invasive plants. Thus we are concerned about the future development direction of plant invasion under the influence of multiple GCFs. The results suggest that apart from precipitation increase and nitrogen deposition (which synergistically promoted plant performance) all other GCFs combinations may not enhance plant performance or relative competitiveness. Moreover, the data suggests that under combined increased precipitation and nitrogen deposition the performance responses of native and invasive plants were similar, so invasive plants might not gain higher relative competitiveness. Therefore, although global change is generally believed to be beneficial to invasive plants, most factors may not greatly promote plant invasions. The patterns in plant responses to combined GCFs serve as important indicators for predicting future ecosystem change. This synthesis of plant responses can form the baseline for studying the performance of common plants (i.e. non-invasive species) under the scenario of future global change. In fact, the interaction of GCFs on regular plants has also become a hot issue to be further studied due to its complexity (Gray and Brady, 2016; van der Kooi et al., 2016; Piao et al., 2019). There are several major caveats to the interpretation of the results. Most of the experiments were conducted over the short-term (either only over one growing season or aborted prior to flowering and senescence), or did not gauge plant responses in survivorship. The identity of these species likely has a strong influence on individual results, and those tend to be common species or successful invaders (i.e., rare endemic species and introduced but non-invasive species are not represented in the data). These factors reduce the generality of the results and long-term studies are needed to give clearer indications to plant responses under global change.

The results indicate that plants with improved performance due to global change factors do not necessarily have stronger competitiveness and vice versa. Therefore, it may be important to assess both plant performance and competitiveness. In addition, in the process of literature collection and analysis, the data selection criteria is based on the criteria of previous studies (Kuebbing and Nuñez, 2016; Liu et al., 2017; Yue et al., 2019). The analysis of other variables or combinations was not possible due to limitation in data availability. For instance, there were only few studies testing three or more GCFs, which is understandable given the significant increase in samples necessary with every new factor included. However, these studies may be necessary to test how different factors interact, especially under the background of warming. In addition, we analyzed the performance and competitiveness response of invasion and native plants in different plant groups (such as the availability of nitrogen fixation capacity, life cycle, and functional groups) to GCFs (**Figure S3**). Unfortunately, due to the low availability of data whether responses differ among plant functional groups could not be assessed in this study (e.g., for the combination between nitrogen deposition and elevated CO_2_, there were only 2 studies and both are of non-nitrogen fixers. For precipitation increase and nitrogen, there were only 4 observations for annual species and none for other life histories. For the combination between drought and nitrogen, 14 observations were of grasses or herbs and only 4 for shrubs). According to the results of this study, the combined effects of multiple global change factors are mostly non-additive. Therefore, results of single factors are likely moderated by the interaction with other factors. Considering the differences in species and experimental conditions among experiments, it is necessary to test the relevant theories through the systematic design of multiple factor experiments.

## Conclusions

Our research revealed that studies focusing on the additive effects of multiple global change factors on the performance and competitiveness of native and invasive plants are uncommon. Moreover, the combined effects of multiple factors on native and invasive plants cannot simply be inferred from the effects of single factors individually. Invasive plants tend to be advantaged over native plants, especially under a change in water regime and CO_2_ enrichment, but this advantage will not be magnified with multiple global change factors because of the accumulation of factors. Our results suggest that although plant invasions will become more severe and extensive under global changes, they are not always uncontrollable.

## Data availability statement

The original contributions presented in the study are included in the article/**Supplementary Material**. Further inquiries can be directed to the corresponding authors.

## Author contributions

BY wrote the manuscript, conceptualized the study, and analyzed the data. MC collated the data and analyzed the data. YD collated the data. GR, CW, ZD and SR reviewed and edited drafts of the paper. GL commented on the manuscript. JW conceptualized the study, reviewed, and edited drafts of the manuscript. DD conceptualized the study, acquired funding, and provided supervision. All authors contributed to the article and approved the submitted version.

## Acknowledgments

We acknowledge financial support by the National Natural Science Foundation of China (32071521, 32001087, 32271587), Carbon peak and carbon neutrality technology innovation foundation of Jiangsu Province (BK20220030), Natural Science Foundation of Jiangsu Province (BK20211321), Jiangsu province young scientist's grant (BK20200905), the Jiangsu Collaborative Innovation Center of Technology and Material of Water Treatment., and Jiangsu Province and Education Ministry Co-sponsored Synergistic Innovation Center of Modern Agricultural Equipment. Susan Rutherford and Justin SH Wan are supported by the Jiangsu University Research Foundation Fund (20JDG055, 20JDG056).

## Conflict of interest

The authors declare that the research was conducted in the absence of any commercial or financial relationships that could be construed as a potential conflict of interest.

## Publisher’s note

All claims expressed in this article are solely those of the authors and do not necessarily represent those of their affiliated organizations, or those of the publisher, the editors and the reviewers. Any product that may be evaluated in this article, or claim that may be made by its manufacturer, is not guaranteed or endorsed by the publisher.

## Supplementary material

Supplementary Figure 1 Flowchart of the literature screening process detailing the number of studies excluded at each stage up to the final number of studies included in the analyses.

Supplementary Figure 2 Source location or region of literature **(A)** and study **(B)** used in analysis. The scatter chart in the middle of each figure indicates the number of literature/studies from each country in different years (note that the dot size of the two figures represents the difference of values). The top and right sides of the scatter plot show the total number of literature/studies by year and country, respectively.

Supplementary Figure 3 Effects across GCFs on different plant groups: Nitrogen fixation **(A)**, Life cycle **(B)**, functional group **(C)**, and performance response **(D)**. Values indicate the means with 95% confidence intervals (CIs), and original sample size numbers for native and invasive plants are shown behind right the dots. The * to the left of the dots indicate that CI does not overlap with 0, which means that the GCF has a significant impact on plants. Positive effects are where the CI is greater than 0 and negative effects are where the CI is less than 0.
