# Supplementary figures and images for: Influence of multiple global change drivers on plant invasion: Additive effects are uncommon
Source: Front Plant Sci. 2022 Nov 14;13:1020621. doi: 10.3389/fpls.2022.1020621 (PMC9702074; doi:10.3389/fpls.2022.1020621)

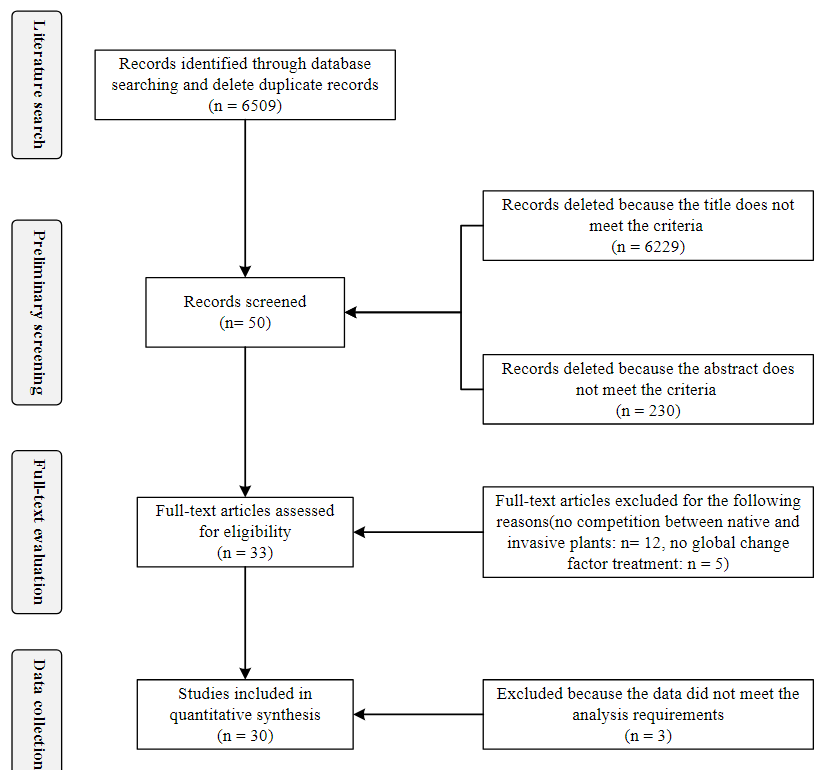

Supplement: Supplementary Figure 1 — Flowchart of the literature screening process detailing the number of studies excluded at each stage up to the final number of studies included in the analyses. [file Image_1.tiff]

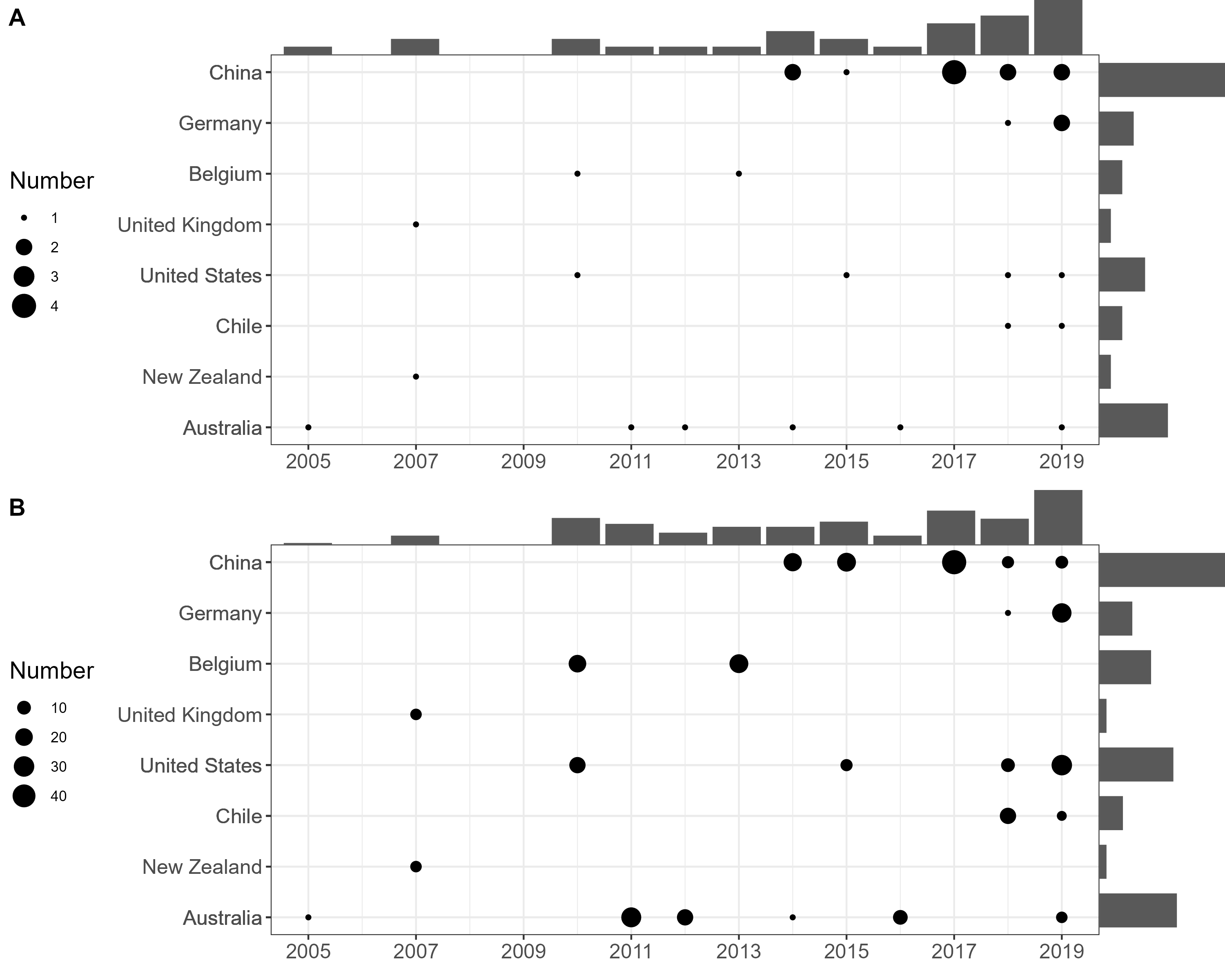

Supplement: Supplementary Figure 2 — Source location or region of literature (A) and study (B) used in analysis. The scatter chart in the middle of each figure indicates the number of literature/studies from each country in different years (note that the dot size of the two figures represents the difference of values). The top and right sides of the scatter plot show the total number of literature/studies by year and country, respectively. [file Image_2.tiff]

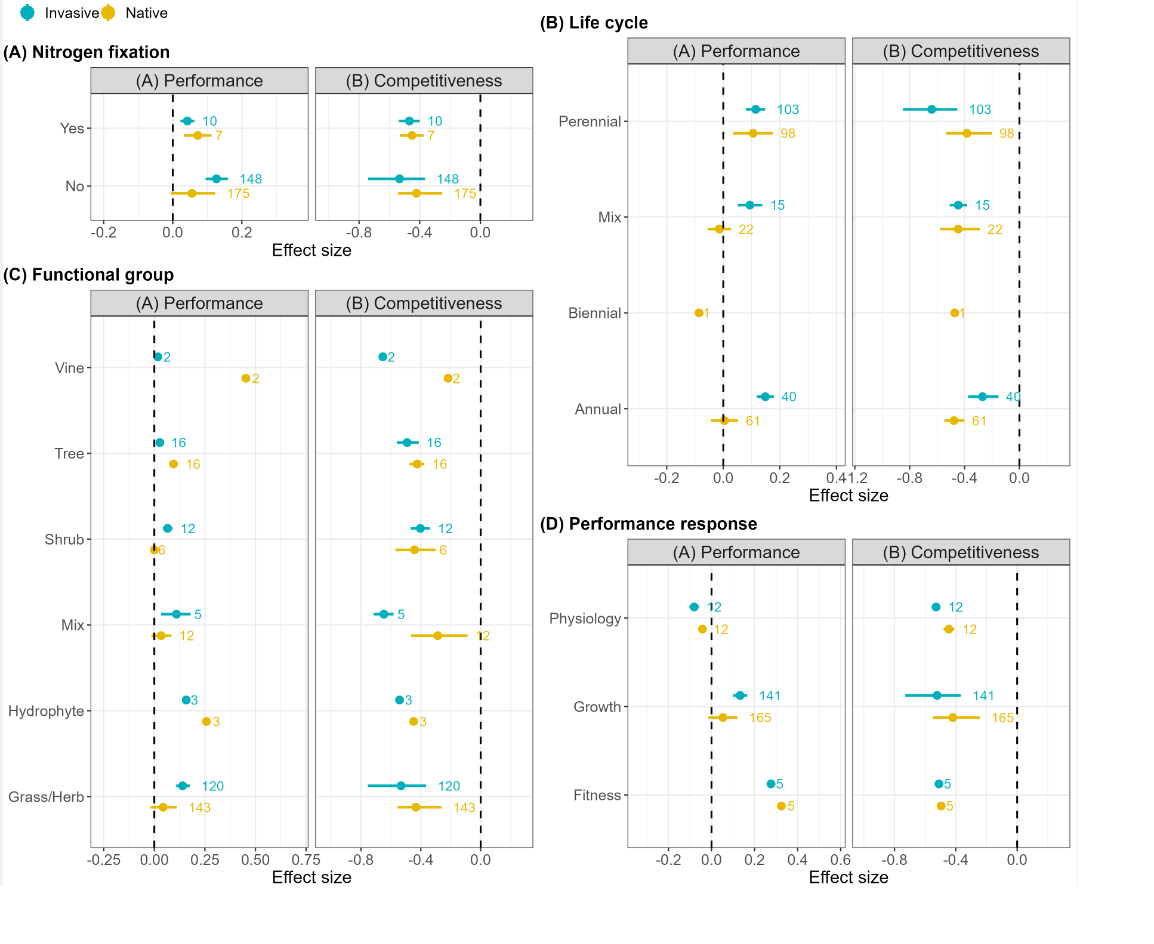

Supplement: Supplementary Figure 3 — Effects across GCFs on different plant groups: Nitrogen fixation (A), Life cycle (B), functional group (C), and performance response (D). Values indicate the means with 95% confidence intervals (CIs), and original sample size numbers for native and invasive plants are shown behind right the dots. The * to the left of the dots indicate that CI does not overlap with 0, which means that the GCF has a significant impact on plants. Positive effects are where the CI is greater than 0 and negative effects are where the CI is less than 0. [file Image_3.tif]
