# Supplementary material for: Influence of multiple global change drivers on plant invasion: Additive effects are uncommon
Source: Front Plant Sci. 2022 Nov 14;13:1020621. doi: 10.3389/fpls.2022.1020621 (PMC9702074; doi:10.3389/fpls.2022.1020621)
Supplement: Supplementary file 4 [file Table_1.docx]

**S1 List of studies included in the** **analysis on effects of global change factors on plant invasion.**

[1] Haeuser, E., Dawson, W., Kleunen, M., & Buckley, Y. (2018). Introduced garden plants are strong competitors of native and alien residents under simulated climate change. *J Ecol*, 107(3), 1328-1342. doi:10.1111/1365-2745.13101

[2] Bueno, A., Pritsch, K., & Simon, J. (2019). Species-Specific Outcome in the Competition for Nitrogen Between Invasive and Native Tree Seedlings. *Front Plant Sci*, 10, 337. doi:10.3389/fpls.2019.00337

[3] Valliere, J. M. (2019). Tradeoffs between growth rate and water-use efficiency in seedlings of native perennials but not invasive annuals. *Plant Ecol*, 220(3), 361-369. doi:10.1007/s11258-019-00919-y

[4] Yu, H., Shen, N., Yu, S., Yu, D., & Liu, C. (2018). Responses of the native species *Sparganium angustifolium* and the invasive species *Egeria densa* to warming and interspecific competition. *Plos One*, 13(6), e0199478. doi:10.1371/journal.pone.0199478

[5] Cavieres, L. A., Sanhueza, A. K., Torres-Mellado, G., & Casanova-Katny, A. (2017). Competition between native *Antarctic vascular* plants and invasive *Poa annua* changes with temperature and soil nitrogen availability. *Biol Invasions*, 20(6), 1597-1610. doi:10.1007/s10530-017-1650-7

[6] Liu, G., Yang, Y. B., & Zhu, Z. H. (2018). Elevated nitrogen allows the weak invasive plant *Galinsoga quadriradiata* to become more vigorous with respect to inter-specific competition. *Sci Rep*, 8(1), 3136. doi:10.1038/s41598-018-21546-z

[7] Legault, R., 2nd, Zogg, G. P., & Travis, S. E. (2018). Competitive interactions between native *Spartina alterniflora* and non-native *Phragmites australis* depend on nutrient loading and temperature. *Plos One*, 13(2), e0192234. doi:10.1371/journal.pone.0192234

[8] Wang, C., Zhou, J., Liu, J., & Jiang, K. (2017). Differences in functional traits between invasive and native Amaranthus species under different forms of N deposition. *Naturwissenschaften*, 104, 59. doi:10.1007/s00114-017-1482-4

[9] Huang, Q. Q., Shen, Y. D., Li, X. X., Fan, Z. W., Li, S. L., & Liu, Y. (2017). Performance of the invasive *Eupatorium catarium* and *Ageratum conyzoides* in comparison with a common native plant under varying levels of light and moisture. *Weed Biol Manag*, 17(2), 112-121. doi:10.1111/wbm.12125

[10] Wu, H., Ismail, M., & Ding, J. (2017). Global warming increases the interspecific competitiveness of the invasive plant alligator weed, *Alternanthera philoxeroides*. *Sci Total Environ*, 575, 1415-1422. doi:10.1016/j.scitotenv.2016.09.226

[11] Wang, A. O., Jiang, X.-X., Zhang, Q.-Q., Zhou, J., Li, H.-L., Luo, F.-L., et al. (2015). Nitrogen addition increases intraspecific competition in the invasive wetland plant *Alternanthera philoxeroides*, but not in its native congenerAlternanthera sessilis. *Plant Spec Biol*, 30(3), 176-183. doi:10.1111/1442-1984.12048

[12] Luo, Y. J., Guo, W. H., Yuan, Y. F., Liu, J., Du, N., & Wang, R. Q. (2014). Increased nitrogen deposition alleviated the competitive effects of the introduced invasive plant *Robinia pseudoacacia* on the native tree *Quercus acutissima*. *Plant Soil*, 385(1-2), 63-75. doi:10.1007/s11104-014-2227-1

[13] Tooth, I. M., & Leishman, M. R. (2013). Elevated carbon dioxide and fire reduce biomass of native grass species when grown in competition with invasive exotic grasses in a savanna experimental system. *Biol Invasions*, 16(2), 257-268. doi:10.1007/s10530-013-0448-5

[14] Verlinden, M., Van Kerkhove, A., & Nijs, I. (2013). Effects of experimental climate warming and associated soil drought on the competition between three highly invasive West European alien plant species and native counterparts. *Plant Ecol*, 214(2), 243-254. doi:10.1007/s11258-012-0163-9

[15] Mason, T. J., French, K., Russell, K., & Acosta, A. (2012). Are competitive effects of native species on an invader mediated by water availability? *J Veg Sci*, 23(4), 657-666. doi:10.1111/j.1654-1103.2012.01393.x

[16] Manea, A., & Leishman, M. R. (2011). Competitive interactions between native and invasive exotic plant species are altered under elevated carbon dioxide. *Oecologia*, 165(3), 735-744. doi:10.1007/s00442-010-1765-3

[17] Rao, L. E., & Allen, E. B. (2010). Combined effects of precipitation and nitrogen deposition on native and invasive winter annual production in California deserts. *Oecologia*, 162(4), 1035-1046. doi:10.1007/s00442-009-1516-5

[18] Hely, S. E. L., & Roxburgh, S. H. (2005). The interactive effects of elevated CO_2_, temperature and initial size on growth and competition between a native C3 and an invasive C3 grass. *Plant Ecol*, 177(1), 85-98. doi:10.1007/s11258-005-2247-2

[19] Williams, A. L., Wills, K. E., Janes, J. K., Vander Schoor, J. K., Newton, P. C., & Hovenden, M. J. (2007). Warming and free-air CO_2_ enrichment alter demographics in four co-occurring grassland species. *New Phytol*, 176(2), 365-374. doi:10.1111/j.1469-8137.2007.02170.x

[20] Liu, Y., Zhang, X., Kleunen, M., & Cooke, J. (2018). Increases and fluctuations in nutrient availability do not promote dominance of alien plants in synthetic communities of common natives. *Funct Ecol*, 32(11), 2594-2604. doi:10.1111/1365-2435.13199

[21] Wei, C. Q., Tang, S. C., Pan, Y. M., & Li, X. Q. (2017). Plastic responses of invasive *Bidens frondosa* to water and nitrogen addition. *Nord J Bot*, 35(2), 232-239. doi:10.1111/njb.01331

[22] Manea, A., Sloane, D. R., & Leishman, M. R. (2016). Reductions in native grass biomass associated with drought facilitates the invasion of an exotic grass into a model grassland system. *Oecologia*, 181(1), 175-183. doi:10.1007/s00442-016-3553-1

[23] Ashbacher, A. C., & Cleland, E. E. (2015). Native and exotic plant species show differential growth but similar functional trait responses to experimental rainfall. *Ecosphere*, 6(11), 1-14. doi:10.1890/Es15-00059.1

[24] Li, H. L., Ning, L., Alpert, P., Li, J. M., & Yu, F. H. (2014). Responses to simulated nitrogen deposition in invasive and native or non-invasive clonal plants in China. *Plant Ecol*, 215(12), 1483-1492. doi:10.1007/s11258-014-0408-x

[25] Verlinden, M., & Nijs, I. (2010). Alien plant species favoured over congeneric natives under experimental climate warming in temperate Belgian climate. *Biol Invasions*, 12(8), 2777-2787. doi:10.1007/s10530-009-9683-1

[26] Gufu, G. D., Manea, A., & Leishman, M. R. (2019). Experimental evidence that CO_2_ and nutrient enrichment do not mediate interactions between a native and an exotic free-floating macrophyte. *Hydrobiologia*, 846(1), 75-85. doi:10.1007/s10750-019-04053-8

[27] Molina-Montenegro, M. A., Bergstrom, D. M., Chwedorzewska, K. J., Convey, P., & Chown, S. L. (2019). Increasing impacts by Antarctica's most widespread invasive plant species as result of direct competition with native vascular plants. *Neobiota*, (51), 19-40. doi:10.3897/neobiota.51.37250

[28] Wang, N., & Chen, H. (2019). Increased nitrogen deposition increased the competitive effects of the invasive plant *Aegilops tauschii* on wheat. *Acta Physiologiae Plantarum*, 41(10). doi:10.1007/s11738-019-2968-9

[29] Wang, Y. J., Chen, D., Yan, R., Yu, F. H., & van Kleunen, M. (2019). Invasive alien clonal plants are competitively superior over co-occurring native clonal plants. *Perspect Plant Ecol*, 40, 125484. doi:10.1016/j.ppees.2019.125484

[30] Bradford, M. A., Schumacher, H. B., Catovsky, S., Eggers, T., Newingtion, J. E., & Tordoff, G. M. (2007). Impacts of invasive plant species on riparian plant assemblages: interactions with elevated atmospheric carbon dioxide and nitrogen deposition. *Oecologia*, 152(4), 791-803. doi:10.1007/s00442-007-0697-z
