# Supplementary material for: Influence of multiple global change drivers on plant invasion: Additive effects are uncommon
Source: Front Plant Sci. 2022 Nov 14;13:1020621. doi: 10.3389/fpls.2022.1020621 (PMC9702074; doi:10.3389/fpls.2022.1020621)
Supplement: Supplementary file 5 [file Table_2.docx]

**Table S1** List of species included in comprehensive analysis on influence of multiple global change drivers on plant invasion. The study number corresponds to the references provided below the table.

| **Species** | **Family** | **Native continent(s)** | **Growth Habit** | **Life History** | **Study number** |
| --- | --- | --- | --- | --- | --- |
| *Tetragonia tetragonoides* | Aizoaceae | Asia, Oceania | Herbs | Annual | 15 |
| *Carpobrotus glaucescens* | Aizoaceae | Oceania | Herbs | Perennial | 15 |
| *Alternanthera philoxeroides* | Amaranthaceae | S. America | Herbs | Perennial | 10, 11, 24, 29 |
| *Alternanthera sessilis* | Amaranthaceae | Asia | Herbs | Perennial | 11, 24 |
| *Amaranthus retroflexus* | Amaranthaceae | Americas | Herbs | Annual | 8 |
| *Amaranthus tricolor* | Amaranthaceae | Asia | Herbs | Annual | 8 |
| *Chenopodium candolleanum*  */Rhagodia candolleana* | Amaranthaceae | Oceania | Shrub | Perennial | 15 |
| *Digitaria sanguinalis* | Amaranthaceae | Asia | Herbs | Annual | 10, 16 |
| *Allium schoenoprasum* | Amaryllidaceae | Asia, Europe, N. America | Herbs | Perennial | 1 |
| *Hydrocotyle peduncularis* | Araliaceae | Asia, Oceania | Herbs | Perennial | 15 |
| *Hydrocotyle sibthorpioides* | Araliaceae | Asia | Herbs | Perennial | 29 |
| *Hydrocotyle vulgaris* | Araliaceae | Europe, North America | Herbs | Perennial | 29 |
| *Araujia sericifolia* | Asclepiadaceae | Oceania | Vine | Perennial | 16 |
| *Achillea millefolium* | Asteraceae | Asia, Europe, N. America | Herbs | Perennial | 1 |
| *Ageratum conyzoides* | Asteraceae | S. America | Herbs | Annual | 9 |
| *Artemisia californica* | Asteraceae | N. America | Shrub | Perennial | 3 |
| *Artemisia verlotiorum* | Asteraceae | China | Herbs | Perennial | 25 |
| *Artemisia vulgaris* | Asteraceae | Europe, Asia, N. Africa, Alaska | Herbs | Perennial | 25 |
| *Bidens frondosa* | Asteraceae | N. America | Herbs | Annual | 21, 25 |
| *Bidens pilosa* | Asteraceae | Americas | Herbs | Annual | 16 |
| *Bidens tripartita* | Asteraceae | Eurasia, N. Africa, N. America | Herbs | Annual | 21, 25 |
| *Centaurea melitensis* | Asteraceae | Mediterranean | Herbs | Annual | 3 |
| *Chrysanthemoides monilifera* | Asteraceae | S. Africa | Shrub | Perennial | 15 |
| *Cirsium arvense* | Asteraceae | Europe, Africa, N. Asia | Herbs | Perennial | 14 |
| *Conyza bonariensis* | Asteraceae | S. America | Herbs | Annual | 16 |
| *Encelia californica* | Asteraceae | N. America | Shrub | Perennial | 3 |
| *Eupatorium catarium* | Asteraceae | S. America | Herbs | Annual | 9 |
| *Galinsoga quadriradiata* | Asteraceae | N. America | Herbs | Annual | 6 |
| *Heteropappus hispidus* | Asteraceae | Eurasia | Herbs | Annual | 6 |
| *Hypochaeris radicata* | Asteraceae | Europe | Herbs | Perennial | 19 |
| *Leontodon autumnalis* | Asteraceae | Asia, Europe, N. America | Herbs | Perennial | 1 |
| *Leontodon taraxacoides* | Asteraceae | Europe, N. Africa | Herbs | Perennial | 19 |
| *Leucanthemum vulgare* | Asteraceae | Asia, Europe | Herbs | Perennial | 1 |
| *Ozothamnus diosmifolius* | Asteraceae | Oceania | Shrub | Perennial | 15 |
| *Rudbeckia triloba* | Asteraceae | N. America | Herbs | Perennial | 1 |
| *Senecio inaequidens* | Asteraceae | Africa | Herbs | Perennial | 14, 25 |
| *Senecio jacobaea* | Asteraceae | Europe, Asia | Herbs | Perennial | 25 |
| *Sigesbeckia orientalis* | Asteraceae | Africa, Asia, Oceania | Herbs | Annual | 16 |
| *Solidago gigantea* | Asteraceae | N. America | Herbs | Perennial | 1, 14, 25 |
| *Solidago virgaurea* | Asteraceae | Europe | Herbs |  | 25 |
| *Sonchus oleraceus* | Asteraceae | Eurasia | Herbs | Annual | 6 |
| *Sphagneticola calendulacea*  */Wedelia chinensis* | Asteraceae | Asia | Grass | Perennial | 24 |
| *Sphagneticola chinensis* | Asteraceae | Asia | Herbs | Perennial | 29 |
| *Sphagneticola trilobata*  */Wedelia trilobata* | Asteraceae | N. America & S. America | Grass | Perennial | 24 |
| *Vernonia cinerea* | Asteraceae | Asia | Herbs | Annual | 9 |
| *Sphagneticola trilobata* | Asteraceae | North and South, America | Herbs | Perennial | 29 |
| *Impatiens glandulifera* | Balsaminaceae | Asia | Herbs | Annual | 25, 30 |
| *Impatiens noli-tangere* | Balsaminaceae | Europe, Asia and N. America | Herbs | Annual | 25 |
| *Amsinckia Tessellata* | Boraginaceae | N. America | Herbs | Annual | 17 |
| *Barbarea stricta* | Brassicaceae | Europe, Asia | Herbs |  | 25 |
| *Barbarea vulgaris* | Brassicaceae | Europe | Herbs | Biennial | 25 |
| *Diplotaxis tenuifolia* | Brassicaceae | Africa, Asia, Europe | Herbs | Perennial | 1 |
| *Hesperis matronalis* | Brassicaceae | Europe | Herbs | Biennial  /Perennial | 1 |
| *Hirschfeldia incana* | Brassicaceae | Mediterranean | Herbs | Annual | 3 |
| *Lepidium campestre* | Brassicaceae | Europe | Herbs | Annual | 25 |
| *Lepidium draba* | Brassicaceae | S. Europe, SW. Asia, N. Africa | Herbs | Perennial | 25 |
| *Platycodon grandiflorus* | Campanulaceae | Asia | Herbs | Perennial | 1 |
| *Cerastium arvense* | Caryophyllaceae | Europe, N. America, S. America | Herbs | Perennial | 25 |
| *Cerastium tomentosum* | Caryophyllaceae | Asia, Europe | Herbs | Perennial | 1, 25 |
| *Colobanthus quitensis* | Caryophyllaceae | Antarctica | Herbs | Perennial | 5, 27 |
| *Silene dioica* | Caryophyllaceae | Europe | Herbs | Biennial  /Perennial | 1 |
| *Silene flos-cuculi* | Caryophyllaceae | Europe | Herbs | Perennial | 1 |
| *Silene vulgaris* | Caryophyllaceae | Europe | Herbs | Perennial | 1 |
| *Atriplex semibaccata* | Chenopodiaceae | Oceania | Herbs | Perennial | 16 |
| *Chenopodium robertianum*  */Einadia hastata* | Chenopodiaceae | Oceania | Herbs | Perennial | 16 |
| *Calystegia sepium* | Convolvulaceae | Europe | Herbs | Perennial | 30 |
| *Calystegia soldanella* | Convolvulaceae | Europe, Asia, Oceania | Herbs | Perennial | 15 |
| *Convolvulus erubescens* | Convolvulaceae | Oceania | Vine | Perennial | 16 |
| *Ipomoea cairica* | Convolvulaceae | Africa | Herbs | Perennial | 24 |
| *Ipomoea pes-caprae* | Convolvulaceae | Pantropical | Herbs | Perennial | 25, 24 |
| *Acacia implexa* | Fabaceae | Oceania | Herbs | Perennial | 16 |
| *Acacia parramattensis* | Fabaceae | Oceania | Herbs | Perennial | 16 |
| *Acmispon glaber* | Fabaceae | N. America | Shrub | Perennial | 3 |
| *Glycine microphylla* | Fabaceae | Oceania | Vine | Perennial | 16 |
| *Lathyrus latifolius* | Fabaceae | S.Europe & E. Europe | Herbs | Perennial | 25 |
| *Lathyrus pratensis* | Fabaceae | Europe | Herbs | Perennial | 25 |
| *Lotus corniculatus* | Fabaceae | Africa, Asia, Europe | Herbs | Perennial | 1 |
| *Lupinus polyphyllus* | Fabaceae | N. America | Herbs | Perennial | 1 |
| *Robinia pseudoacacia* | Fabaceae | N. America | Tree | Perennial | 2, 12 |
| *Trifolium pratense* | Fabaceae | Africa, Asia, Europe | Herbs | Biennial  /Perennial | 1 |
| *Trifolium repens* | Fabaceae | Europe, North Africa | Herbs | Perennial | 29 |
| *Fagus sylvatica* | Fagaceae | Europe | Tree | Perennial | 2 |
| *Quercus acutissima* | Fagaceae | Asia | Herbs | Perennial | 12 |
| *Quercus robur* | Fagaceae | Europe | Tree | Perennial | 2 |
| *Erodium cicutarium* | Geraniaceae | Mediterranean | Herbs | Annual | 3 |
| *Egeria densa* | Hydrocharitaceae | S. America | Herbs | Perennial | 4 |
| *Iris domestica* | Iridaceae | Asia | Herbs | Perennial | 1 |
| *Monarda fistulosa* | Lamiaceae | N. America | Herbs | Perennial | 1 |
| *Monarda punctata* | Lamiaceae | N. America | Herbs | Perennial | 1 |
| *Plectranthus parviflorus* | Lamiaceae | Oceania | Herbs | Annual | 16 |
| *Salvia mellifera* | Lamiaceae | N. America | Shrub | Perennial | 3 |
| *Sida rhombifolia* | Malvaceae | Americas | Herbs | Perennial | 16 |
| *Leptospermum laevigatum* | Myrtaceae | Oceania | Shrub | Perennial | 15 |
| *Ligustrum sinense* | Oleaceae | Asia | Herbs | Perennial | 16 |
| *Olea ferruginea* | Oleaceae | Asia | Herbs | Perennial | 16 |
| *Epilobium ciliatum* | Onagraceae | Asia, N. America, S. America | Herbs | Perennial | 1 |
| *Epilobium hirsutum* | Onagraceae | Europe, Africa, Asia | Herbs | Perennial | 14, 30 |
| *Ludwigia adscendens* | Onagraceae | Asia | Herbs | Perennial | 29 |
| *Oenothera biennis* | Onagraceae | N. America | Herbs | Biennial  /Perennial | 1 |
| *Oxalis corniculat* | Oxalidaceae | Asia | Herbs | Perennial | 29 |
| *Mimulus guttatus* | Phrymaceae | N. America | Herbs | Perennial | 1 |
| *Breynia oblongifolia* | Phyllanthaceae | Oceania | Shrub | Perennial | 15 |
| *Phytolacca octandra* | Phytolaccaceae | N. America | Herbs | Perennial | 16 |
| *Pinus sylvestris* | Pinaceae | Europe | Tree | Perennial | 2 |
| *Antirrhinum majus* | Plantaginaceae | Europe | Herbs | Annual  /Perennial | 1 |
| *Plantago lanceolata* | Plantaginaceae | Europe | Herbs | Perennial | 14 |
| *Veronica persica* | Plantaginaceae | Asia, so. Europe | Herbs | Annual | 1 |
| *Aegilops tauschii* | Poaceae | Asia | Grass | Annual | 28 |
| *Andropogon gayanus* | Poaceae | Africa | Herbs | Perennial | 13 |
| *Austrodanthonia caespitosa* | Poaceae | Oceania | Grass | Perennial | 19 |
| *Austrodanthonia eriantha* | Poaceae | Oceania | Grass | Perennial | 18 |
| *Avena fatua* | Poaceae | Eurasia | Herbs | Annual | 3 |
| *Axonopus compressus* | Poaceae | N. America & S. America | Grass | Perennial | 24 |
| *Bothriochloa macra* | Poaceae | Oceania | Grass | Perennial | 16, 22 |
| *Bromus diandrus* | Poaceae | Mediterranean | Herbs | Annual | 3 |
| *Bromus madritensis* | Poaceae | Europe | Grass | Annual | 17 |
| *Cenchrus ciliaris* | Poaceae | Africa | Herbs | Perennial | 13 |
| *Cenchrus polystachion* | Poaceae | Africa | Herbs | Perennial | 13 |
| *Chloris gayana* | Poaceae | Africa | Grass | Perennial | 16 |
| *Chloris truncata* | Poaceae | Oceania | Grass | Perennial | 16 |
| *Cynodon dactylon* | Poaceae | Eastern hemisphere | Grass | Annual | 15 |
| *Deschampsia antarctica* | Poaceae | Antarctica | Herbs | Perennial | 5, 27 |
| *Dichanthium sericeum* | Poaceae | Oceania | Grass | Perennial | 16 |
| *Ehrharta erecta* | Poaceae | S. Africa | Grass | Perennial | 22 |
| *Eragostis brownii* | Poaceae | Oceania | Grass | Perennial | 16 |
| *Eragostis curvula* | Poaceae | N. America | Grass | Perennial | 16 |
| *Eriachne triseta* | Poaceae | Oceania | Herbs | Perennial | 13 |
| *Heteropogon contortus* | Poaceae | Africa | Herbs | Perennial | 13 |
| *Heteropogon triticeus* | Poaceae | Asia, Oceania | Herbs | Perennial | 13 |
| *Imperata cylindrica* | Poaceae | Europe, Asia, Oceania | Grass | Perennial | 15 |
| *Ischaemum triticeum* | Poaceae | Oceania | Grass | Annual | 15 |
| *Microlaena stipoides* | Poaceae | Oceania | Grass | Perennial | 22 |
| *Panicum brevifolium* | Poaceae | Asia, Africa, Brazil | Grass | Annual | 24 |
| *Panicum repens* | Poaceae | Brazil | Grass | Perennial | 24 |
| *Paspalum conjugatum* | Poaceae | Tropical America | Grass | Perennial | 24 |
| *Paspalum dilatatum* | Poaceae | S. America | Grass | Perennial | 16, 29 |
| *Paspalum distichum* | Poaceae | N. America & S. America | Grass | Perennial | 24 |
| *Paspalum orbiculare* | Poaceae | Asia | Grass | Perennial | 29 |
| *Paspalum vaginatum* | Poaceae | Americas | Grass | Perennial | 15 |
| *Phragmites australis* | Poaceae | Eurasia | Herbs | Perennial | 7 |
| *Poa annua* | Poaceae | Eurasia | Herbs | Perennial | 5, 27 |
| *Poa trivialis* | Poaceae | Europe | Herbs | Perennial | 30 |
| *Rytidosperma racemosum*  */Austrodanthonia racemosa* | Poaceae | Australia | Grass | Perennial | 22 |
| *Sorghum halepense* | Poaceae | Europe, Africa, Asia | Grass | Perennial | 16 |
| *Spartina alterniflora* | Poaceae | N. America | Herbs | Perennial | 7 |
| *Spinifex sericeus* | Poaceae | Oceania | Grass | Annual | 15 |
| *Sporobolus creber* | Poaceae | Oceania | Grass | Perennial | 16 |
| *Sporobolus indicus* | Poaceae | Oceania | Grass | Perennial | 16 |
| *Stenotaphrum helferi* | Poaceae | Asia | Grass | Perennial | 24 |
| *Stipa pulchra*  */Nassella pulchra* | Poaceae | N. America | Herb | Perennial | 3 |
| *Themeda australis* | Poaceae | Oceania | Grass | Perennial | 16, 22 |
| *Themeda triandra* | Poaceae | Africa, Asia, Oceania | Grass | Perennial | 19 |
| *Triticum aestivum* | Poaceae |  | Grass | Annual | 28 |
| *Vulpia myuros* | Poaceae | Eurasia | Grass | Annual | 18 |
| *Zoysia macrantha* | Poaceae | Oceania | Grass | Perennial | 15 |
| *Gilia tricolor* | Polemoniaceae | N. America | Herbs | Annual  /perennial | 1 |
| *Fallopia japonica* | Polygonaceae | Asia | Herbs | Perennial | 30 |
| *Fallopia japonica*  */Reynoutria japonica* | Polygonaceae | Asia | Herbs | Perennial | 14 |
| *Persicaria capitata* | Polygonaceae | Asia | Herbs | Perennial | 1 |
| *Polygonum cuspidatum* | Polygonaceae | Asia | Herbs | Perennial | 30 |
| *Rumex acetosa* | Polygonaceae | Europe, Asia | Herbs | Perennial | 25 |
| *Rumex obtusifolius* | Polygonaceae | Europe | Herbs | Perennial | 30 |
| *Rumex sagittatus*  */Acetosa sagittata* | Polygonaceae | Africa | Vine | Perennial | 16 |
| *Rumex scutatus* | Polygonaceae | S. Europe, SW. Asia, N. Africa | Herbs |  | 25 |
| *Banksia integrifolia* | Proteaceae | Oceania | Shrub | Perennial | 15 |
| *Ranunculus repens* | Ranunculaceae | Europe, Asia | Herbs | Perennial | 30 |
| *Prunus serotina* | Rosaceae | Americas | Tree | Perennial | 2 |
| *Correa alba* | Rutaceae | Oceania | Shrub | Perennial | 15 |
| *Azolla filiculoides* | Salviniaceae | Asia, Oceania, America | Hydrophyte | Perennial | 26 |
| *Salvinia molesta* | Salviniaceae | S. America | Hydrophyte | Perennial | 26 |
| *Myoporum boninense* | Scrophulariaceae | Oceania | Shrub | Perennial | 15 |
| *Sparganium angustifolium* | Typhaceae | Asia | Herbs | Perennial | 4 |
| *Urtica dioica* | Urticaceae | Europe | Herbs | Perennial | 30 |
| *Verbena rigida* | Verbenaceae | S. America | Herbs | Perennial | 1 |
| *Viola tricolor* | Violaceae | Europe | Herbs | Perennial | 1 |
| *Nepeta racemosa* | Labiatae | Asia | Herbs | Perennial | 1 |

[1] Haeuser, E., Dawson, W., Kleunen, M., & Buckley, Y. (2018). Introduced garden plants are strong competitors of native and alien residents under simulated climate change. J Ecol, 107(3), 1328-1342. doi:10.1111/1365-2745.13101

[2] Bueno, A., Pritsch, K., & Simon, J. (2019). Species-Specific Outcome in the Competition for Nitrogen Between Invasive and Native Tree Seedlings. Front Plant Sci, 10, 337. doi:10.3389/fpls.2019.00337

[3] Valliere, J. M. (2019). Tradeoffs between growth rate and water-use efficiency in seedlings of native perennials but not invasive annuals. Plant Ecol, 220(3), 361-369. doi:10.1007/s11258-019-00919-y

[4] Yu, H., Shen, N., Yu, S., Yu, D., & Liu, C. (2018). Responses of the native species Sparganium angustifolium and the invasive species Egeria densa to warming and interspecific competition. Plos One, 13(6), e0199478. doi:10.1371/journal.pone.0199478

[5] Cavieres, L. A., Sanhueza, A. K., Torres-Mellado, G., & Casanova-Katny, A. (2017). Competition between native Antarctic vascular plants and invasive Poa annua changes with temperature and soil nitrogen availability. Biol Invasions, 20(6), 1597-1610. doi:10.1007/s10530-017-1650-7

[6] Liu, G., Yang, Y. B., & Zhu, Z. H. (2018). Elevated nitrogen allows the weak invasive plant Galinsoga quadriradiata to become more vigorous with respect to inter-specific competition. Sci Rep, 8(1), 3136. doi:10.1038/s41598-018-21546-z

[7] Legault, R., 2nd, Zogg, G. P., & Travis, S. E. (2018). Competitive interactions between native Spartina alterniflora and non-native Phragmites australis depend on nutrient loading and temperature. Plos One, 13(2), e0192234. doi:10.1371/journal.pone.0192234

[8] Wang, C., Zhou, J., Liu, J., & Jiang, K. (2017). Differences in functional traits between invasive and native Amaranthus species under different forms of N deposition. Naturwissenschaften, 104, 59. doi:10.1007/s00114-017-1482-4

[9] Huang, Q. Q., Shen, Y. D., Li, X. X., Fan, Z. W., Li, S. L., & Liu, Y. (2017). Performance of the invasive Eupatorium catarium and Ageratum conyzoides in comparison with a common native plant under varying levels of light and moisture. Weed Biol Manag, 17(2), 112-121. doi:10.1111/wbm.12125

[10] Wu, H., Ismail, M., & Ding, J. (2017). Global warming increases the interspecific competitiveness of the invasive plant alligator weed, Alternanthera philoxeroides. Sci Total Environ, 575, 1415-1422. doi:10.1016/j.scitotenv.2016.09.226

[11] Wang, A. O., Jiang, X.-X., Zhang, Q.-Q., Zhou, J., Li, H.-L., Luo, F.-L., et al. (2015). Nitrogen addition increases intraspecific competition in the invasive wetland plantAlternanthera philoxeroides, but not in its native congenerAlternanthera sessilis. Plant Spec Biol, 30(3), 176-183. doi:10.1111/1442-1984.12048

[12] Luo, Y. J., Guo, W. H., Yuan, Y. F., Liu, J., Du, N., & Wang, R. Q. (2014). Increased nitrogen deposition alleviated the competitive effects of the introduced invasive plant Robinia pseudoacacia on the native tree Quercus acutissima. Plant Soil, 385(1-2), 63-75. doi:10.1007/s11104-014-2227-1

[13] Tooth, I. M., & Leishman, M. R. (2013). Elevated carbon dioxide and fire reduce biomass of native grass species when grown in competition with invasive exotic grasses in a savanna experimental system. Biol Invasions, 16(2), 257-268. doi:10.1007/s10530-013-0448-5

[14] Verlinden, M., Van Kerkhove, A., & Nijs, I. (2013). Effects of experimental climate warming and associated soil drought on the competition between three highly invasive West European alien plant species and native counterparts. Plant Ecol, 214(2), 243-254. doi:10.1007/s11258-012-0163-9

[15] Mason, T. J., French, K., Russell, K., & Acosta, A. (2012). Are competitive effects of native species on an invader mediated by water availability? J Veg Sci, 23(4), 657-666. doi:10.1111/j.1654-1103.2012.01393.x

[16] Manea, A., & Leishman, M. R. (2011). Competitive interactions between native and invasive exotic plant species are altered under elevated carbon dioxide. Oecologia, 165(3), 735-744. doi:10.1007/s00442-010-1765-3

[17] Rao, L. E., & Allen, E. B. (2010). Combined effects of precipitation and nitrogen deposition on native and invasive winter annual production in California deserts. Oecologia, 162(4), 1035-1046. doi:10.1007/s00442-009-1516-5

[18] Hely, S. E. L., & Roxburgh, S. H. (2005). The interactive effects of elevated CO2, temperature and initial size on growth and competition between a native C3 and an invasive C3 grass. Plant Ecol, 177(1), 85-98. doi:10.1007/s11258-005-2247-2

[19] Williams, A. L., Wills, K. E., Janes, J. K., Vander Schoor, J. K., Newton, P. C., & Hovenden, M. J. (2007). Warming and free-air CO2 enrichment alter demographics in four co-occurring grassland species. New Phytol, 176(2), 365-374. doi:10.1111/j.1469-8137.2007.02170.x

[20] Liu, Y., Zhang, X., Kleunen, M., & Cooke, J. (2018). Increases and fluctuations in nutrient availability do not promote dominance of alien plants in synthetic communities of common natives. Funct Ecol, 32(11), 2594-2604. doi:10.1111/1365-2435.13199

[21] Wei, C. Q., Tang, S. C., Pan, Y. M., & Li, X. Q. (2017). Plastic responses of invasive Bidens frondosa to water and nitrogen addition. Nord J Bot, 35(2), 232-239. doi:10.1111/njb.01331

[22] Manea, A., Sloane, D. R., & Leishman, M. R. (2016). Reductions in native grass biomass associated with drought facilitates the invasion of an exotic grass into a model grassland system. Oecologia, 181(1), 175-183. doi:10.1007/s00442-016-3553-1

[23] Ashbacher, A. C., & Cleland, E. E. (2015). Native and exotic plant species show differential growth but similar functional trait responses to experimental rainfall. Ecosphere, 6(11), 1-14. doi:10.1890/Es15-00059.1

[24] Li, H. L., Ning, L., Alpert, P., Li, J. M., & Yu, F. H. (2014). Responses to simulated nitrogen deposition in invasive and native or non-invasive clonal plants in China. Plant Ecol, 215(12), 1483-1492. doi:10.1007/s11258-014-0408-x

[25] Verlinden, M., & Nijs, I. (2010). Alien plant species favoured over congeneric natives under experimental climate warming in temperate Belgian climate. Biol Invasions, 12(8), 2777-2787. doi:10.1007/s10530-009-9683-1

[26] Gufu, G. D., Manea, A., & Leishman, M. R. (2019). Experimental evidence that CO2 and nutrient enrichment do not mediate interactions between a native and an exotic free-floating macrophyte. Hydrobiologia, 846(1), 75-85. doi:10.1007/s10750-019-04053-8

[27] Molina-Montenegro, M. A., Bergstrom, D. M., Chwedorzewska, K. J., Convey, P., & Chown, S. L. (2019). Increasing impacts by Antarctica's most widespread invasive plant species as result of direct competition with native vascular plants. Neobiota(51), 19-40. doi:10.3897/neobiota.51.37250

[28] Wang, N., & Chen, H. (2019). Increased nitrogen deposition increased the competitive effects of the invasive plant Aegilops tauschii on wheat. Acta Physiologiae Plantarum, 41(10). doi:10.1007/s11738-019-2968-9

[29] Wang, Y. J., Chen, D., Yan, R., Yu, F. H., & van Kleunen, M. (2019). Invasive alien clonal plants are competitively superior over co-occurring native clonal plants. Perspect Plant Ecol, 40, 125484. doi:10.1016/j.ppees.2019.125484

[30] Bradford, M. A., Schumacher, H. B., Catovsky, S., Eggers, T., Newingtion, J. E., & Tordoff, G. M. (2007). Impacts of invasive plant species on riparian plant assemblages: interactions with elevated atmospheric carbon dioxide and nitrogen deposition. Oecologia, 152(4), 791-803. doi:10.1007/s00442-007-0697-z
